# Sex-related differences in dietary phytochemical intake in the population of primary school children in urban setting

**DOI:** 10.3389/fnut.2025.1576803

**Published:** 2025-06-18

**Authors:** Martina Bituh, Ana Ilić, Petra Škorvaga, Ružica Brečić, Irena Colić Barić, Ivana Rumbak

**Affiliations:** ^1^Department of Food Quality Control, Faculty of Food Technology and Biotechnology, University of Zagreb, Zagreb, Croatia; ^2^Department of Marketing, Faculty of Economics and Business, University of Zagreb, Zagreb, Croatia

**Keywords:** carotenoids, childhood, glucosinolates, phytonutrients, phytosterols, polyphenols

## Abstract

**Background:**

The consumption of phytochemicals is known for its positive effects on human health. However, there are no established recommendations for daily intake in European or international guidelines. Given the different dietary patterns in different countries and the changes in dietary trends over time, it is crucial to assess the intake of phytochemicals, especially in children, as inadequate consumption may increase the risk of chronic non-communicable diseases. To address this gap, the present study aims to estimate the daily intake of the four major classes of phytochemicals among schoolchildren in urban areas and to identify the main dietary sources and subclasses that contribute most to their intake.

**Methods:**

Daily intake of the four main classes of phytochemicals and their main dietary sources was assessed in school children from an urban setting (*N* = 195; 8.9 ± 0.4 years). Intake of phytochemicals was estimated from 3-day dietary records analyzed with available composition tables and databases.

**Results:**

The median intake of polyphenols in children was 125.7 mg/day, of carotenoids 3.9 mg/day, of phytosterols 126.1 mg/day and of glucosinolates 2.3 mg/day, with girls having a significantly higher intake of polyphenols. Although fruit and vegetables are the most common sources of phytochemicals in the diet, an increased contribution of sweets and cakes and mixed dishes was observed. Food groups rich in phytochemicals (fruits, vegetables, legumes, nuts and seeds) contributed less than 10% of the daily energy intake.

**Conclusion:**

Although there are no official recommendations for the intake of phytochemicals, the study suggests that children have a low intake of all four main classes of phytochemicals compared to existing data for both children and adults. To improve the intake of phytochemicals, it is necessary to promote the consumption of foods rich in phytochemicals and increase dietary diversity both at home and at school.

## Introduction

In the context of nutrition and human health, the intake of phytochemicals has been recognized for their beneficial effects (1–3). However, there is no recommended daily intake for phytochemicals in the European food standards, the guidelines of the World Health Organization or the guidelines of non-European countries (4–7). The problem of defining recommendations for the intake of phytochemicals is challenging due to their wide chemical diversity, the lack of evidence linking their health effects to the consumption of whole foods rather than isolated compounds and lack of clearly defined biological functions or deficiency syndromes (8). Furthermore, it is difficult to accurately estimate the actual intake of phytochemicals, that may 1 day be linked to health outcomes in epidemiological studies, as there is still a lack of comprehensive and standardized food composition databases containing values for the broad spectrum of phytochemicals. Only a few existing food databases contain the amount of polyphenols in food, such as the USDA Food Database, which contains information on 38 different phytochemicals from the group of carotenoids, phytosterols and polyphenols (9, 10). Even fewer databases contain the content of phytochemicals together with the associated retention factors, one such database is Phenol Explorer (11). Therefore, the evaluation of the intake of phytochemicals is usually limited to an assessment based on the data of individual scientific studies. Furthermore, the bioavailability and accessibility of phytochemicals are strongly influenced by food processing, food matrix and metabolism (1, 12, 13). There is also a lack of epidemiological studies evaluating the intake of phytochemicals and linking it to health outcomes in different life stages, especially in children. To the authors’ knowledge, there are only a few studies that have estimated the intake of phytochemicals, with polyphenols being the most frequently observed main class of phytochemicals (14–22).

Due to different dietary patterns among countries and dietary trends in intake over time (23) it seems important to collect such data, especially in children as low consumption could be a risk of development of chronic non communicable diseases.

To fill this knowledge gap, the present study aims to estimate the daily intake of the four main classes of phytochemicals and to determine which subclasses of each main class of phytochemicals contribute most to their daily intake in schoolchildren from urban settings. In addition, this study aims to evaluate which subclasses of each main class of phytochemicals contribute most to their daily intake. In addition, the present study aims to identify the major dietary contributors to the daily intake of four main classes of phytochemicals.

## Subjects and methods

### Study population and design

This study uses a cross-sectional approach based on data collected as part of a previously conducted prospective longitudinal intervention study carried out over a three-year period (school year 2018/2019 to 2020/2021) in elementary school in Zagreb, Croatia, participating in the “Pilot Project: School Meals and Fruit and Vegetable Intake in Schools With and Without a Garden” (24). For this study, data was extracted from the baseline of the prospective longitudinal intervention study. This study involved school-age children (8.9 ± 0.4 years) from 14 elementary school in the city of Zagreb. The present study was carried on 195 schoolchildren (28.6% of total study population included in the project) which 3-day dietary records were collected. The minimum number of 184 children, 92 per gender, was estimated to detect a group difference with 95% power at a significance of *α* = 0.05 significance (version 3.1.9.2; Heinrich Heine University Dusseldorf, Dusseldorf, Germany). Before the study was conducted, the parents/caregivers gave their written consent for each child to participate in the study.

### Phytochemical data analysis

Phytochemical data were analyzed using dietary food records from three non-consecutive days. Parents/caregivers completed the dietary records accompanied by their children to minimize the bias that may occur when eating outside the home (e.g., at school, playground, friends’ houses, etc.). All food and drink consumed was weighted or given as a household measure if weighting was not possible. For the school meals consumed, the type of meal and the percentage of the portion consumed were recorded so that the quantity of food and drink could be calculated using the meal recipes (given by schools to the research team). After data collection, all recipes and mixed dishes were divided into the individual foods contained in the dish, where possible. Mixed dishes that could not be itemized included, for example, fish sticks, pizza slices, purchased desserts, baked goods, cheese-filled tortellini, etc. and were considered food per se. In addition, all household units were converted to gram/milliliter units. All foods and beverages were categorized into 15 different food groups based on the dietary guidelines for school children and modified to better determine their association with the intake of selected phytochemicals (25). Daily energy intake (kcal) and the relative contribution of different food groups to the daily energy intake were analyzed using software Prehrana (Infosistem, d.d, Zagreb, Croatia). The software contained national composition tables (26) and was supplemented with the nutritional information from the food labels of the foods or the complexed dishes (e.g., pizza from bakery).

The Phenol-Explorer database was used to estimate daily polyphenol intake - total flavonoids, total phenolic acids, stilbenes, lignans and other polyphenols (11). The total daily intake of polyphenols was calculated as the sum of the estimated subclasses of polyphenols. Data were corrected with retention factors when different heat treatment methods were involved (27).

The total daily intake of carotenoids includes the assessment of the daily intake of lutein and zeaxanthin, *β*-carotene and lycopene. Data for *β*-carotene were taken from the national composition tables (26). For the foods for which no amount of carotene was given in the Croatian tables, the values were taken from the US Department of Agriculture database (10). The US Department of Agriculture database was also used to calculate the other subclasses of carotenoids. Data for all subclasses of carotenoids were corrected with retention factors when different methods of thermal processing were used (28).

Data from the study conducted by Witkowska et al. (29) were used to estimate the intake of stigmasterol, campesterol and *β*-sitosterol and data from the USDA databases were used for the intake of brassicasterol, ergosterol and other phytosterols (10). The daily intake of phytosterols was calculated as the sum of the estimated subclassis. Data were corrected using retention factors when different methods of thermal processing were involved (29).

The glucosionlate intake was assessed using data from Steinbrecher and Linseisen (30). Data were corrected using retention factors when different methods of thermal processing were involved (31–33). The uptake of the individual glucosinolates (glucoibervirin, glucoerucin, dehydroerucin, glucoberteroin, glucoiberin, glucoraphanin, glucoraphenin, glucoalyssin, glucoheirolin, glucoerysoline, glucocochlearin, sinigrin, gluconapin, glucobrassicanapin, progoitrin, epiprogoitrin, napoleiferin, glucotropaeolin, gluconasturtiin, sinalbin, glucobarbarin, glucobrassicin, 4-hydroxyglucobrassicin, neoglucobrassicin, 4-methoxyglucobrassicin) was summarized as the intake of aliphatic glucosinolates, indolylglucosinolates and aromatic glucosinolates. The total daily intake of glucosinolates corresponds to the sum of the three glucosinolate subclasses.

### Anthropometric measurements

The anthropometric measurements, body height and body weight, were performed in accordance with the World Health Organization guidelines by trained person from the research team during physical education and health classes while children were wearing light athletic clothing. Both were measured to the nearest 0.1 cm and 0.1 kg using a combined medical digital scale and stadiometer (Seca, type 877–217, Vogel & Halke Gmbh & Co., Hamburg, Germany). Weight status were assessed using the World Health Organization cut-off for the sex-and age-standardized z-score for body mass index (kg/m^2^) (34, 35).

### Statistical analysis

Observed individual means method was used to present daily energy and macronutrient intake (36). The normality of the data distribution was estimated using the Shapiro–Wilk test. Continuous variables (age, energy intake and polyphenol intake) were presented as mean and standard deviation or median and interquartile range, while categorical variables were presented as percentages. The relative contribution of phytochemical subclass to daily intake of four main classes of phytochemical, of food groups to daily intake of energy and four main classes of phytochemical, and of food items to daily intake of four main phytochemical classes were expressed as mean percentage. Differences between boys and girls were test using Students *t*-test for two independent variables or Mann-U-Whitney test. The statistical analysis was performed using IBM SPSS Statistics v. 23.0, released 2015 (IBM SPSS Statistics for Windows, Version 23.0. Armonk, NY, United States: IBM Corp.), with significance set at *p* < 0.05 for all analyzes.

## Results

The study sample consisted of 195 children attending elementary school, of whom 52.3% were boys and 47.7% girls aged 8.9 ± 0.4 years. The majority of the children (75.9%) had a normal weight status according to the World Health Organization cut-offs for body mass index. The overall prevalence of overweight and obesity was 12.6 and 9.9%, respectively. No significant differences in weight status were found between boys and girls.

**Figure 1:**
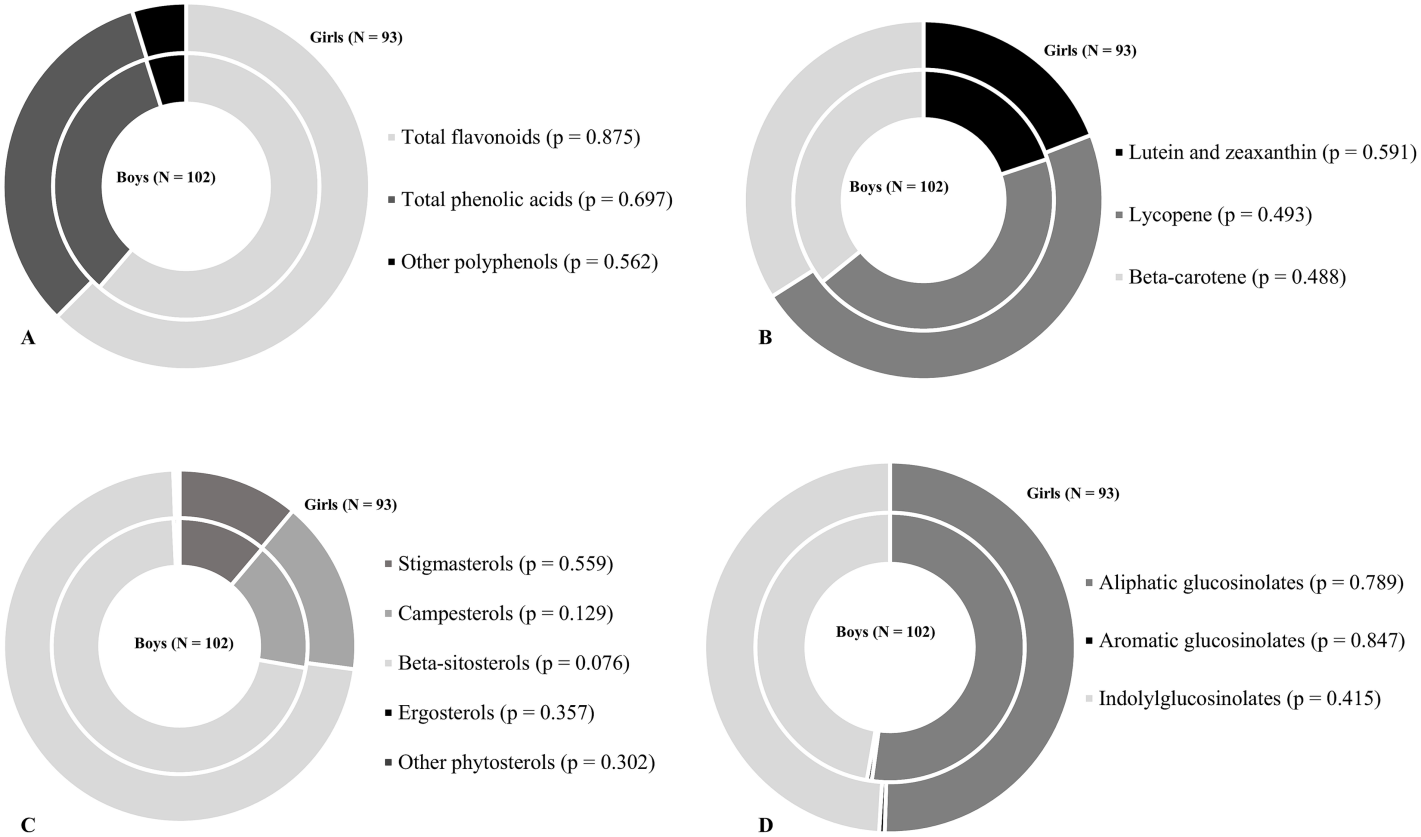
The relative contribution of the phytochemical subclasses to the total intake of selected phytochemicals among boys and girls. **(A)** Contribution to the total polyphenol intake. **(B)** Contribution to the total carotenoid intake. **(C)** Contribution to the total phytosterols intake. **(D)** Contribution to the total glucosinolates intake. The inner circle represents the proportion of phytocemical subclasses in the total of phytocemical in boys, while the outer circle represents the proportion of phytocemical subclasses in the total of phytocemical in girls. The differences between sexes were tested using Mann U-Whitney test or Student’s *t*-test (*p* < 0.05).

Intake of phytochemicals, divided by subclasses are shown in Table 1. No differences were found between boys and girls in the intake of carotenoids, phytosterols and glucosinolates. However, girls had a higher intake of total polyphenols (158.3 mg/day, [81.5–241.5] *vs.* 114.1 mg/day, [64.9–172.3]; *p* = 0.009), total flavonoids (93.6 mg/day, [51.0–155.1] *vs.* 66.7, [36.3–112.8]; *p* = 0.012) and total phenolic acids (42.7 mg/day, [24.6–84.7] *vs.* 33.5, [18.2–58.9]; *p* = 0.034) than the boys. Neither boys nor girls consumed stilbenes, lignans and brasicasterols. Furthermore, there were no differences between the sexes in the relative contribution of the phytochemical subclasses to the daily intake of main phytochemical classes (Figure 1).

**Table 1:** Differences in average daily intake of phytochemicals classes and subclasses between boys and girls.^1^

Phytochemicals (mg/day)	Total (*N* = 195)	Boys (*N* = 102)	Girls (*N* = 93)	*p*-value*
Total polyphenols ^2^	125.8 (73.7–209.9)	114.4 (64.9–172.3)	158.3 (81.5–241.5)	0.009
Total flavonoids	75.9 (41.2–138.4)	66.7 (36.3–112.8)	93.6 (51.0–155.1)	0.012
Total phenolic acids	37.6 (21.5–66.8)	33.5 (18.2–58.9)	42.7 (24.6–84.7)	0.034
Stilbenes	–	–	–	–
Lignans	–	–	–	–
Other polyphenols	2.1 (0.0–7.3)	1.6 (0.0–6.7)	3.3 (0.0–7.4)	0.369
Total carotenoids ^3^	3.9 (2.2–6.9)	3.7 (2.1–6.5)	4.1 (2.5–7.2)	0.333
Beta-carotene	1.2 (0.6–2.1)	1.2 (0.6–2.0)	1.3 (0.6–2.1)	0.714
Lutein and zeaxanthin	0.5 (0.2–1.1)	0.4 (0.2–1.0)	0.5 (0.2–1.1)	0.685
Lycopene	1.5 (0.5–3.4)	1.4 (0.4–2.9)	1.8 (0.6–3.8)	0.217
Total phytosterols ^4^	126.0 (106–1–153.9)	126.3 (108.4–153.6)	124.7 (102.0–156.0)	0.498
Stigmasterols	12.6 (10.0–18.1)	13.7 (10.0–18.6)	12.4 (9.8–17.5)	0.399
Campesterols	20.8 (15.8–25.6)	21.4 (16.9–26.1)	20.2 (15.2–25.4)	0.329
Beta-sitosterols	89.9 (74.3–109.1)	90.4 (77.1–108.6)	89.5 (73.0–109.6)	0.722
Brasicasterols	–	–	–	–
Ergosterols	0.0 (0.0–0.0)	0.0 (0.0–0.0)	0.0 (0.0–0.0)	0.350
Other phytosterols	0.0 (0.0–0.4)	0.0 (0.0–0.4)	0.0 (0.0–0.4)	0.365
Total glucosinolates ^5^	2.3 (0.0–7.4)	1.6 (0.0–6.9)	2.8 (0.0–9.9)	0.237
Aliphatic glucosinolates	1.0 (0.0–4.4)	0.7 (0.0–4.2)	1.4 (0.0–5.6)	0.365
Indolylglucosinolates	1.1 (0.0–3.6)	0.9 (0.0–2.7)	1.3 (0.0–4.9)	0.272
Aromatic glucosinolates	0.0 (0.0–0.0)	0.0 (0.0–0.0)	0.0 (0.0–0.0)	0.548

^1^ All variables are presented a medina (interquartile range). ^2^ Total polyphenols represent the sum of the total flavonoids, total phenolic acids, stilbenes, lignans and other polyphenols. ^3^ Total carotenoids represent sum of the beta-carotene, lutein, zeaxanthin and lycopene. ^4^ Total phytosterols represent the sum of stigmasterols, campesterols, beta-sitosterols, brasicasterols, ergosterols and other phytosterols. ^5^ Total glucosinolates represent the sum of the aliphatic glucosinolates, indolylglucosinolates and aromatic glucosionlates. ^6^ Not observed in children diet. * The differences between sexes were tested using Mann U-Whitney test or Student’s *t*-test (*p* < 0.05).

**Figure 2:**
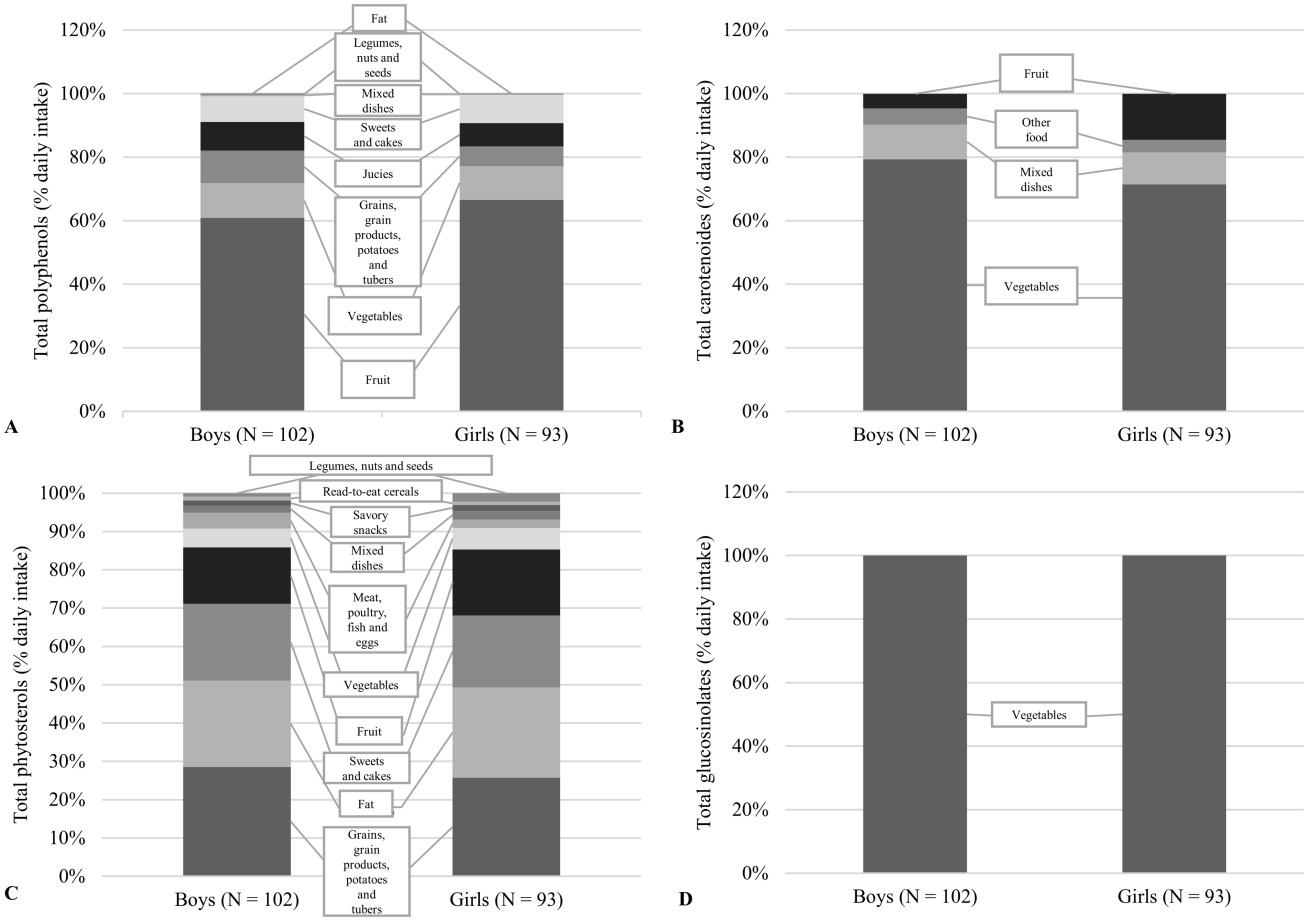
The relative contribution of the food groups to total intake of selected phytochemicals among boys and girls. **(A)** Contribution to the total polyphenol intake. **(B)** Contribution to the total carotenoid intake. **(C)** Contribution to the total phytosterols intake. **(D)** Contribution to the total glucosinolates intake.

The contribution of certain food groups to the daily intake of main phytochemical classes is shown in Figure 2. As expected, for both boys and girls, more than 70% of the daily intake of polyphenols came from the consumption of fruit, followed by vegetables. Among fruit, the polyphenols came mainly from various berries, especially cherries, apples and fruit juices (100% orange juice and apple juice), while among vegetables from onions and potatoes. Vegetables (79% of total carotenoids in boy and 71% in girls) and mixed dishes (11% of total carotenoids in boys and 10% in girls) contribute the most to the daily intake of carotenoids in both sexes. It has been shown that tomatoes and tomato products as well as pizza from the mixed food group are a good source of carotenoids for children. In addition, grains, grain products, potatoes and tubers (29% of total phytosterols in boys and 26% in girls) contribute to the daily intake of phytoestrogens, followed by fats (22% of total phytosterols in boys and 24% in girls), sweets and cakes (20% of total phytosterols in boys and 19% in girls), and fruit (15% of total phytosterols in boys and 17% in girls). The most dominant food source of phytoestrogens was sunflower oil, followed by various types of wheat and wholemeal breads and rolls and chocolate products. Finally, only the vegetable group, *Brassica* vegetables, was found to be a source of daily intake of glucosinolates in both sexes.

## Discussion

The results of the present study provide an overview of the intake of phytochemicals, i.e., the intake of polyphenols, carotenoids, phytosterols and glucosinolates in children living in the urban settings. This study provides valuable results as the intake of phytochemicals in children is rarely observed, especially the intake of phytochemicals other than polyphenols (14–22).

The intake of polyphenols is one of the most extensively studied phytochemical intake in children (14, 15, 18–20, 22). The median daily intake was 125.8 mg/day and thus below the values determined in studies from Italy (226.3 mg/day in winter; 192.3 mg/day in spring), Argentina (412 mg/day), Canada (734.1 mg/day) and Great Britain (boys: 390.1 mg/day; girls: 387.3 mg/day) (14, 19, 20, 22). Among the five polyphenol subclasses, flavonoids accounted for the largest proportion of daily intake (61%), followed by phenolic acids (33%). The relative contribution of the polyphenol subclasses varied from study to study, probably due to differences in age groups, food preferences and regional dietary patterns. For example, flavonoids accounted for up to 80% of polyphenol intake in Italian and British children, while phenolic acids dominated in Argentinian children (77%) and Canadian children had almost equal intakes of flavonoids and phenolic acids (14, 19, 20, 22). These differences may also reflect the primary food sources. In this study, fruits (especially berries and apples) and vegetables contributed the most, while intake of cocoa products was limited and tea or decaffeinated coffee was not consumed. In contrast, other studies have identified cocoa products, tea and mate as important sources of polyphenols (14, 20). In addition, the girls in this study consumed more total polyphenols, flavonoids and phenolic acids than the boys, although their overall dietary habits were similar. This could be due to food preferences, such as the higher consumption of blueberries and cocoa in girls. Similar gender differences in polyphenol intake have also been observed in other populations, often influenced by both energy intake and food choices (22). The intake of stilbenes and lignans was not assessed in the present study, similar to other studies in which their contribution to the total daily intake of polyphenols was either negligible (less than 1%) or not reported at all (14, 19, 20, 22).

Carotenoids are the most researched phytochemicals after polyphenols, as they are considered reliable biomarkers (serum and skin) for fruit and vegetable intake (37, 38), which is one of the biggest challenges for children’s nutrition in this decade (39–42). However, information on the intake of carotenoids in the population of children is lacking (18, 38, 43–46). The median daily intake of carotenoids was 3.9 mg, which consisted of *β*-carotene (1.2 mg), lycopene (1.5 mg), lutein and zeaxanthin (0.5 mg). In comparison, a study conducted in the USA reported higher intake of carotenoids (3.6–8.2 mg/day) in 9–12 year olds (38), while a study conducted in Spain found a lower intake of β-carotene (0.9 mg), a higher intake of lycopene (2.7 mg) and a similar intake of lutein and zeaxanthin (0.6 mg) in 7–9 year olds (47). The methods and databases used varied from study to study, which may explain the differences observed. For example, our study used dietary records for three non-consecutive days, whereas other studies used FFQ or 24-h recall (38, 47). Studies using three 24-h recall have shown significantly lower intake values compared to studies using food frequency questionnaires (44, 47). In addition, the observed differences may also be attributed to the use of different national food composition tables, although both studies included the USDA food database in addition to their national databases and other national published sources. National published sources are very important. For example, fresh tomatoes grown in three different regions of Croatia were shown to have widely varying levels of lycopene (from 1.82 to 11.19 mg/100 g) (48). Lycopene accounted for 44.7% of the total carotenoid intake. The main sources were tomato-based foods, especially school meals (e.g., stews, tomato sauces), which could explain the high lycopene intake. This is consistent with seasonal availability, as the study was conducted in late spring/summer when fresh tomatoes are more commonly consumed.

Pyhtosterols or plant sterols are structurally similar to cholesterol and intake of 1–3 g per day can lower cholesterol levels in adults and children (49, 50). However, a typical Western diet can provide between 150 and 400 mg of sterols per day (49). Dietary sources of sterols include seeds, vegetable fats and oils and nuts. As, plant sterols have a low bioavailability, a higher intake of plant sterols can be achieved by consuming fortified products or supplements (50, 51). The median daily intake was 126.0 mg, slightly lower than in the Flemish preschool children (46). The Flemish preschool children consumed an average of 175–188 mg of plant sterols per day, with the boys having a higher intake (46). In contrast to a study conducted among Flemish preschool children, which found that 21% of participants consumed plant sterol fortified foods resulting in an average intake of 0.70 g/day and a maximum intake of 2.10 g/day, none of the children in our study consumed plant sterol-fortified foods as part of their regular diet. *β*-sitosterols accounted for the largest proportion (>70%) of the total intake of plant sterols, primarily due to their presence in sunflower and olive oil—the main sources of fat in the children’s diet. The food groups contributing most to the total daily intake of plant sterols were cereals, cereal products, potatoes and tubers, fats and sweets and cakes. This is consistent with an earlier study of Belgian children in which similar food groups contributed the most to phytosterol intake (46).

An additional contribution of this study is the estimation of glucosinolate intake, which has not yet been investigated in children. The median daily intake of glucosinolates was 2.3 mg. In comparison, the median intake in adults in Spain was also 2.32 mg/day, but in Germany it was significantly higher (14 mg/day) (30, 52). It is noteworthy that 38.9% of children did not consume cruciferous vegetables, which are the main source of glucosinalate in our diet, during the study period. Similar observations were made in the adult population, where 20% of adults did not consume cruciferous vegetables (52). White cabbage was the main dietary source of glucosinolates and was often served in school meals as salad or stew. Other cruciferous vegetables (kale, broccoli, cauliflower and Brussels sprouts) were also consumed more frequently at school than at home. This confirms previous findings that school meals can increase the intake of dark green vegetables (53). While in adults the main sources of glucosinolates come from broccoli, cabbage, Brussels sprouts and cauliflower (30, 52), children may avoid these vegetables because of their bitter and pungent taste (54, 55). However, children’s food preferences can be modified by education, repeated exposure and preparation methods (56–61). Aliphatic glucosinolates (51.3%) and indolyl glucosinolates (48.2%) accounted for half of the total intake of glucosinaolates in the present study, whereas aromatic glucosinolates were hardly present in the diet (0.5%). The quantitatively most important glucosinolates in children were glucobrasincin, sinergin and glucoiberin.

Although there are no specific reference values for phytochemicals in the diet, the national dietary guidelines (25) aims to ensure an adequate intake of different food groups and consequently important nutrients, including phytochemicals. Despite a slightly lower energy intake compared to the recommendations, the children in this study followed a similar dietary pattern compared to other studies. The increased consumption of sweets and snacks and the lower consumption of fruit and vegetables was striking. It was found that the food groups rich in phytochemicals (fruit, vegetables, pulses, nuts and seeds) contributed less than 10% to the daily energy intake (Supplementary Figure 1).

### Strengths and limitations

Although the study provides valuable results on the intake of phytochemicals in school-age children, the results should be interpreted with caution. The results of the study should not be generalized to the Croatian child population, as it included only a sample of children from urban settings. Measuring food and beverage consumption in school-aged children can be challenging as they may eat multiple meals outside the home. However, in the present study, food and beverage consumption was estimated using 3-day dietary records kept jointly by parents and children to minimize potential bias and represent strength of this study. In addition, the research team was familiar with the recipes of the school meals. The study was conducted in late spring/summer, which may affect the availability of fruits and vegetables, which can consequently affect the amount and type of phytochemicals and their dietary sources. As there is no comprehensive database for estimating the different classes of phytochemicals, with the exception of polyphenols, different sources of data on the amount of phytochemicals in food were used to ensure the accuracy and completeness of the data and the intake of phytochemicals in children. The intake of phytochemicals could also be underestimated, as no data for phytochemicals is available for some types of processed food which contribute on average 38.1% of their daily energy intake from ultra-processed foods and 15.3% from processed foods (53).

## Conclusion

This study provides new insights into the quality of children’s diets with regard to the intake of phytochemicals. In addition to the intake of polyphenols, it also evaluates the intake of carotenoids, phytosterols and glucosinolates, for which there is few or no research on children’s diets. Although there is no recommendation for the intake of phytochemicals, the results of the study indicate that children have a low intake of all four classes of phytochemicals compared to existing studies on children and adults. The results suggest that the consumption of foods that are good sources of phytochemicals needs to be increased in children. In addition, it would be beneficial to increase the variety of foods available for meal preparation, both at home and at school, to achieve greater diversity in the intake of phytonutrients subclasses. Future studies in this area should focus on linking phytonutrient intake to children’s health status. They should also address the motivations that can influence increased intake, including food preferences and changes in children’s environments.

## Data Availability

The raw data supporting the conclusions of this article will be made available by the authors, without undue reservation.

## Supplementary material

SUPPLEMENTARY FIGURE 1 The relative contribution of the food groups to daily energy intake among boys and girls. The differences between sexes were tested using Mann U-Whitney test or Student *t*-test (*p* < 0.05).
