# Supplementary figures and images for: Sex-related differences in dietary phytochemical intake in the population of primary school children in urban setting
Source: Front Nutr. 2025 Jun 18;12:1576803. doi: 10.3389/fnut.2025.1576803 (PMC12213441; doi:10.3389/fnut.2025.1576803)

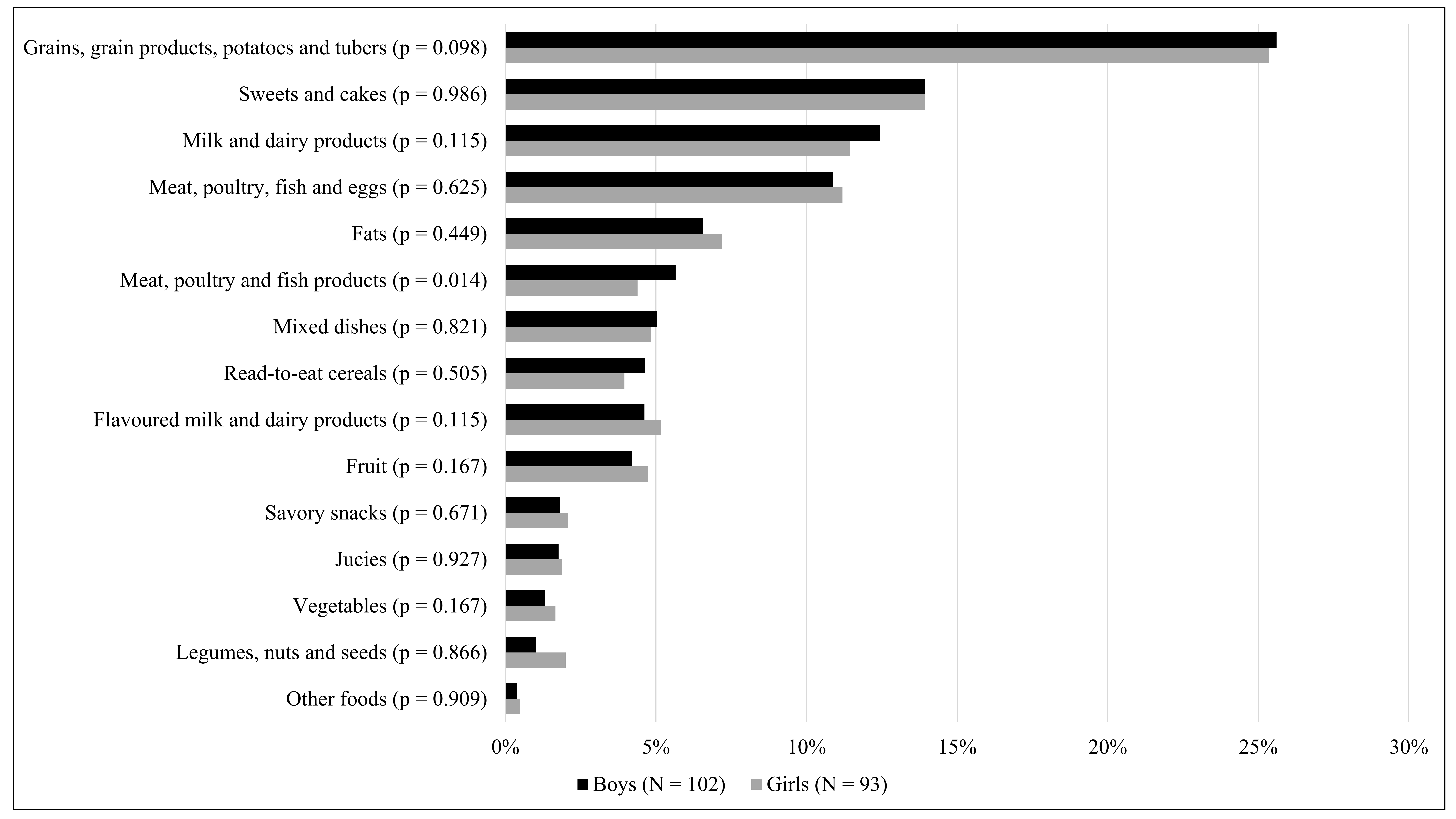

Supplement: SUPPLEMENTARY FIGURE 1 — The relative contribution of the food groups to daily energy intake among boys and girls. The differences between sexes were tested using Mann U-Whitney test or Student t-test (p < 0.05). [file Image_1.tif]
